# Cytogenetic Characteristics of New Monosomic Stocks of Cotton (*Gossypium hirsutum* L.)

**DOI:** 10.4061/2011/273642

**Published:** 2011-03-20

**Authors:** Marina F. Sanamyan, Julia E. Petlyakova, Elnora A. Sharipova, Ibrokhim Y. Abdurakhmonov

**Affiliations:** ^1^Cotton Genetics Laboratory, National University of Uzbekistan, Tashkent, Vuzgorodok 100174, Uzbekistan; ^2^Center of Genomic Technologies, Institute of Genetics and Plant Experimental Biology, Academy of Sciences of Uzbekistan, Yuqori Yuz, Qibray Region, Tashkent 111226, Uzbekistan

## Abstract

The use of aneuploid lines significantly increases the effectiveness of molecular-genetic analysis and the development of superior quality breeding lines via substitutions by alien chromosomes. To date, however, a complete set of aneuploid series for each cotton chromosome is not available. Here, we present the development of a monosomic stock collection of cotton (*Gossypium hirsutum* L.) from Uzbekistan, including the origin of 92 primary monosomics, meiotic metaphase-I analysis, study of tetrads of microspores, pollen fertility, and monosomic transmission rates for some monosomic lines. We report desynaptic effects of some monosomes detected both in parental and daughter monosomics, a positive role of interchanges in translocation heterozygous monosomics due to selective advantages of gametes with deficiency and a simultaneous interchange, pollen fertility variation, and strong differences in transmission rates. This monosomic cotton collection, developed using single genome background, will be useful for future breeding, genetic, cytogenetic, and molecular-genetic investigations of the cotton genome.

## 1. Introduction

The cultivated *Gossypium spp*. (cotton) is an important, natural fiber crop as well as an important source of food, feed, fuel, and other products of significant economic value. The worldwide economic impact of the cotton industry is estimated to be *∼*$500 billion/yr with an annual utilization of ~115 million bales or ~27 million metric tons of cotton fiber [1–3]. Being one of the major cotton producing and exporting countries worldwide, Uzbekistan produces annually 3.5 to 4 million tons of raw cotton fiber and exports cotton fiber valued at ~$0.9 to 1.2 billion. Therefore, development of genetic resources for cotton has been a priority of cotton science in Uzbekistan. Consequently, over the past century, one of the largest cotton germplasm collections, including isogenic, inbred lines, recombinant inbred lines (RIL), elite AD allotetraploid varieties (*Gossypium hirsutum* L. and *Gossypium barbadense* L.), monosomic, and translocation lines along with wild, primitive, and extant representatives of the A- to K-genome groups, has been collected from all over the world, curated, and developed in the Cotton Research Institutes of Uzbekistan [4].

Cultivated allotetraploid cotton, *G. hirsutum *(2*n* = 52), is tolerant to the loss of individual chromosomes or their arms. For many years, efforts toward cotton monosome discovery among the varietal, hybrid, and irradiated populations of cotton as well as among cytogenetic lines were carried out in the USA [5–9]. Up to 1985, monosomes for 15 of 26 nonhomologous chromosomes had been isolated and identified [10]. Since then, in spite of great efforts, there has been minimal success in isolating the rest of 11 monosomes using radiation techniques. A new monosome for chromosome 23 was revealed and identified by meiotic fluorescence in situ hybridization (FISH) in the progeny of interspecific cross in the USA [11], and recently another new monosome for chromosome 21 in cotton was reported [12].

In Uzbekistan, independent investigations on creation of cytogenetic lines in *G. hirsutum *using numerous chromosome aberrant radiomutant plants have been carried out [13–15]. As a result, unlike other multiple-genotype-derived cytogenetic collections for cotton, a new set of monosomic and translocation lines have been developed from the common genetic background of the highly inbred line L-458 (*G. hirsutum*). Some of these monosomes have been characterized with new cytogenetic and biomorphologic characteristics. Previously, we reported our initial study on the origin of 67 primary monosomics obtained from M_1_ and M_2_ generations after irradiation [13]. Later, we reported on the cytology of 3 desynaptic parental cotton plants and their 11 monosomic progenies as well as chromosome transmission rates in 26 monosomics families [14]. However, a detailed characterization of our entire monosomic collection has not been done. Here, we report cytogenetic features for 92 primary monosomics including new monosomics detected in M_3_ generation, newly developed monosomic lines from these 92 primary monosomics, 4 novel desynaptic parental cotton plants and their 4 monosomic progenies, and 19 new monosomic families. We also report new information for 16 of 26 previously reported [14] monosomic families.

## 2. Materials and Methods

### 2.1. Plant Materials

Monosomic lines were developed in a common genetic background of the highly inbred line L-458 *G. hirsutum *L. obtained through multiple generations of self-pollination (F_20_) of 108—F variety. Since all of monosomics were isolated from a common genetic background, some differences observed among them can be attributed to differences in their monosomes. Most of the monosomics were isolated after irradiation of seeds by thermal neutrons or pollen *γ*-irradiation directly in M_1_, M_2_, and M_3_ generations and higher generations of M_4_ to M_6_ were obtained by self-pollination in subsequent years for further genetic analysis. Other monosomic plants were detected in the desynaptic or translocation heterozygote progenies.

### 2.2. Irradiation Techniques

Two types of irradiation, thermal neutron irradiation of seeds and pollen *γ*-irradiation of L-458 line, were used. Irradiation of seeds with thermal neutrons was carried out at the biological channel reactor VVR-SM (Institute of Nuclear Physics of Academy of Science of Republic of Uzbekistan, Tashkent, Uzbekistan). Doses of 15, 25, 27, and 35 Gy were used. For pollen irradiation, mature pollen of L-458 were given *γ*-ray treatment of 10, 15, 20, and 25 Gy (Co^60^, Central Asian Institute of Silk, Tashkent, Uzbekistan) and thereafter were used for pollinating the flowers that were emasculated and enclosed in parchment bags to prevent cross pollination.

### 2.3. Growing Conditions

All M_1_ plants as well as some M_2_ plant families with multiple seeds were grown under field conditions. All the progeny of abnormal plants with a few seeds from M_2_ and M_3_ generations, seeds of desynaptic plants, monosomic translocation heterozygotes, and selfed or outcrossed monosomic plants were germinated on moist filter paper in petridishes at 28°C. Plants were then transplanted into plastic pots with soil. All seedlings were transplanted to greenhouse land soil, and when the first true leaves emerged, transplants were immediately irrigated.

### 2.4. Cytological Analyses

For studies of chromosome pairing at metaphase-I (MI) of meiosis, flower buds were fixed in the morning, after the removal of calyx and corolla, in a solution of 96% alcohol and acetic acid (7 : 3). Buds were kept at room temperature for 3 days then immersed in fresh fixative and stored at 4°C. For cytological preparations, buds were rinsed in tap water before being examined for meiotic associations in the pollen mother cells (PMCs) using the iron acetocarmine squash technique [13–17]. Analyses of chromosomal changes were carried out on the basis of MI associations at the first meiosis of originally isolated monosomic plants (M_1_–M_3_). In monosomic plants and their hybrids, the sizes of univalents were regularly estimated in each subsequent higher generation to check for univalent shifts. The development of PMCs was examined at the tetrad stage for each plant. The meiotic index was calculated as the percentage of normal tetrads in a total sporad [18], and pollen fertility was estimated by acetocarmine staining.

### 2.5. Transmission of the Monosomes

Transmission of the monosomes in M_4_–M_6_ progenies was studied in selfed or outcrossed populations of the monosomic plants. We studied transmission of monosomes in 33 selfed and 12 outcrossed progenies that are detailed in Table 4. All cytological observations were made using Biomed (Leica, Heerburg, Switzerland) or Laboval (Carl Zeiss, Germany) microscopes. Monosomics were numbered in detection order (Mo1–Mo92), and lines were maintained vegetatively in the greenhouse of National University of Uzbekistan.

## 3. Results and Discussion

### 3.1. Origin of the Cotton Primary Monosomics

**Figure 1:**
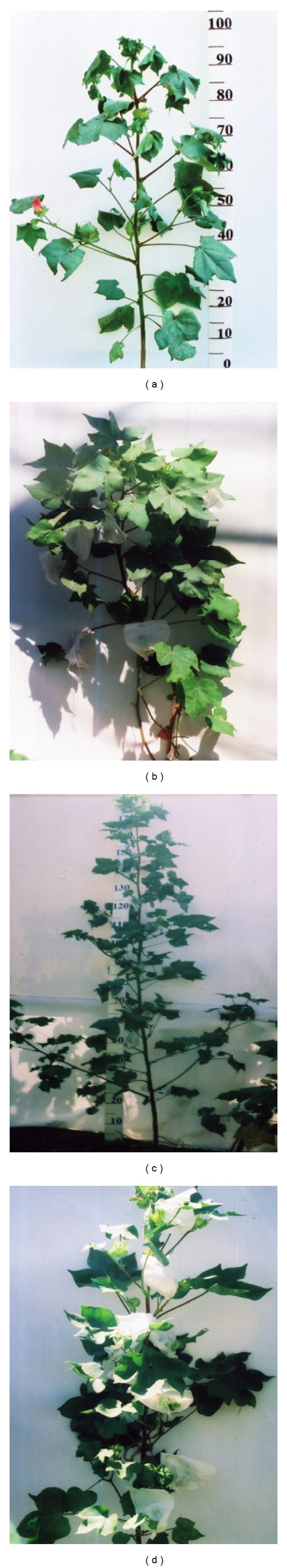
Examples of monosomic plants along with original parental line: (a) control plant L-458; (b) Mo71; (c) Mo80; (d) Mo16. Meiotic configuration of these plants was shown in Figure 2.

Between 1987 and 2007, we developed a total of 92 *G. hirsutum* primary monosomics from the common genetic background of the highly inbred line L-458 (*G. hirsutum*) after irradiation of seeds by thermal neutrons or pollen *γ*-irradiation directly in M_1_, M_2_, and M_3_ generations (for some examples, see Figure 1). Most of them (74 of 92) arose from the two irradiation types directly in M_1_, M_2_, and M_3_ generations (Table 1). The remaining 18 monosomic plants resulted from chromosome aberrant progenies (desynapsis and interchanges, Table 2). Most of the primary monosomics (34) were induced in the M_1_ generation as a result of pollen *γ*-irradiation by doses of 10, 15, 20, and 25 Gy (Table 1). Seven of the monosomic plants had simultaneously independent chromosome interchanges. More than 70% of M_1_ primary monosomics (25 of 34) were induced by high doses of pollen irradiation (20–25 Gy) (Table 1). The number of monosomics detected declined in subsequent generations (24 and 6, resp.), and one M_3_ monosomic (Mo54) also displayed heterozygous translocation.

Similar analyses of cotton plants from seed irradiation with thermal neutrons at doses of 15, 25, 27, and 35 Gy revealed fewer primary monosomics. There were only 10 plants from three generations studied after irradiation; moreover, four of them were also interchanged heterozygotes. In the M_1_ generation, there were only 4 chromosome-deficient plants, 3 of them from the 15 Gy dose and one from the 35 Gy dose. Similarly, 4 monosomic plants were isolated in M_2_ generation in all 4 doses and two monosomic plants were identified from M_3_. 

Previous results demonstrated that pollen *γ*-irradiation with doses of 20 and 25 Gy was the most effective to isolate many chromosome deficient plants [16, 17]. Combined results showed that in M_1_, M_2_, and M_3_ generations, there were 39 (60.9%) monosomic plants. These results do not correspond with the data of Galen and Endrizzi [7] who observed more monosomic plants in lower doses (4 Gy) in comparison with higher doses (12 Gy). 

In addition to traditional radiation-induced cotton monosomics, we used the desynaptic effects which have been found to be a useful source of aneuploidy in other crops. Although desynaptic plants in different crops are usually sterile or show extremely low fertility, the desynaptic cotton plant 1063/6_3_-13 had semisterile pollen due to different numbers of unpaired univalents (from 2 to 28) in PMCs. This desynapsis level may be regarded as intermediate. Another five desynaptic plants were characterized with weak desynapsis level and formed from 2 to 12 univalents. As a result, 16 primary monosomics were isolated from the progenies of 6 desynaptic plants and one unexamined plant from the desynaptic plant progeny (Table 2). 

All the initial desynaptic plants differed by their number of unpaired chromosomes (from 2 to 28 univalents). Disruptions in unpaired chromosome disjunction led to a random univalent distribution between the cell division poles, forming numerous tetrads with micronuclei (to 13.42 ± 0.87%), lowering of meiotic index (to 75.07 ± 1.11% in 356/8 desynaptic plant), and pollen fertility reduction to semisterility (61.35 ± 2.43%). Meiotic index is a normal tetrad percentage and an indicator of meiotic stability proposed by Love [18] for evaluation of meiosis in wheat. We observed that pollen fertility varied among the flowers on the same plant (from 2.61 ± 1.27% to 91.81 ± 1.18% in 179/2 desynaptic plant; Table 2).

It should be noted that the use of an asynaptic genotype in tobacco [19] and desynaptic effect in wheat *Triticum aestivum *L. nulli—3B allowed complete monosomic series to be developed [20, 21] in comparison with cotton [22]. Similar genotype detection in cotton should facilitate the discovery of *de novo* monosomes. Study of desynaptic progenies revealed one unique desynaptic plant (356/8). This plant produced monosomics in high frequency with a small size of univalents and strong phenotypic differences, suggesting monosomy for different chromosomes of cotton genome. Previously, we identified two new monosomics (Mo30 and Mo67) using desynaptic cotton genotype [13].

### 3.2. Meiotic Metaphase-I in Cotton Primary Monosomics

Meiotic metaphase-I analysis of 92 cotton primary monosomics revealed modal chromosome pairing with 25 bivalents and one univalent in 38 plants. Forty-nine monosomic plants were characterized with the presence of additional univalents. Thus, in 32 monosomics, the formation of three univalents in some PMCs was observed due to lack of pairing of single pair of chromosomes. Three monosomics formed five univalents in some PMCs suggesting the absence of pairing in two chromosome pairs. Another five monosomics were characterized with the presence of unpaired chromosomes in 20–30% PMCs. In 8 chromosome-deficient plants, a strong desynaptic effect was detected as they formed from 3 to 11 univalents in 40–60% PMCs studied. In the monosomic plant Mo52, detected in M_2_ generation after pollen *γ*-irradiation, none of the studied PMCs revealed normal chromosome pairing because we noted 3 to 15 univalents.

 Results reported on wheat [21], tobacco [19], and Egyptian Henbane [23] indicate that there is a partial desynaptic effect in some of their monosomes. Monosomic analysis established the presence of the main genes slowing homologous pairing in wheat chromosomes 2B and 3B of the Avrora variety [24]. Chromosome pairing reduction in cotton monosomics with desynapsis could be caused by specific influence of some genes as reported previously [25]. Because the above-mentioned monosomics were detected after irradiation, they might have desynaptic gene mutations, occurring independently of monosomy. In the USA Cotton Cytogenetic Collection, monosomes were also isolated from the partially desynaptic plant progenies [5, 26, 27]; however, these phenomena were not fully characterized. In spite of a lack of reporting on chromosome pairing in monosomics from the USA Cytogenetic Collection [28], it is known that 6 monosomes were isolated from the progenies of other aneuploids as a result of univalent shifts [27]. These were due to disturbances of chromosome behavior in parental monosomics. 

In seven of our monosomes (Mo6, Mo7, Mo19, Mo30, Mo56, Mo61, and Mo62), besides univalents and bivalents, rare trivalents (from 0.04 ± 0.04 to 0.12 ± 0.06 in average per cell) formed at metaphase-I of meiosis. This suggested association of univalents with two homeologous chromosomes. Moreover, two of them (Mo56 and Mo61) were also characterized with additional univalents. The other 12 cotton primary monosomics showed quadrivalent associations with different frequencies suggesting heterozygosity for their translocations. Although we cannot address this with our present data, meiotic associations of univalents with other chromosomes could occasionally be due to homology, or small translocations or duplications, and should be carefully interpreted.

Preliminary evaluation of subgenome assignment showed that translocations in two monosomic plants (Mo9; Mo22) might have been of the A_t_-genome because of their large quadrivalents. Three other monosomics had small quadrivalents (Mo1, Mo54, and Mo63) that apparently originated from the D_t_-genome. The remaining chromosome-deficient plants had quadrivalents of medium size, and their subgenome localization should be determined by genome analysis using the D-genome diploid plants. Ten monosomics with simultaneous translocation heterozygosity were characterized with low quadrivalent frequency, and only two of them (Mo23 and Mo24) had high quadrivalent frequencies (to 0.60 ± 0.13 in average per cell). Besides, 5 monosomics (Mo8, Mo9, Mo23, Mo24, and Mo61) had also additional univalents.

**Figure 2:**
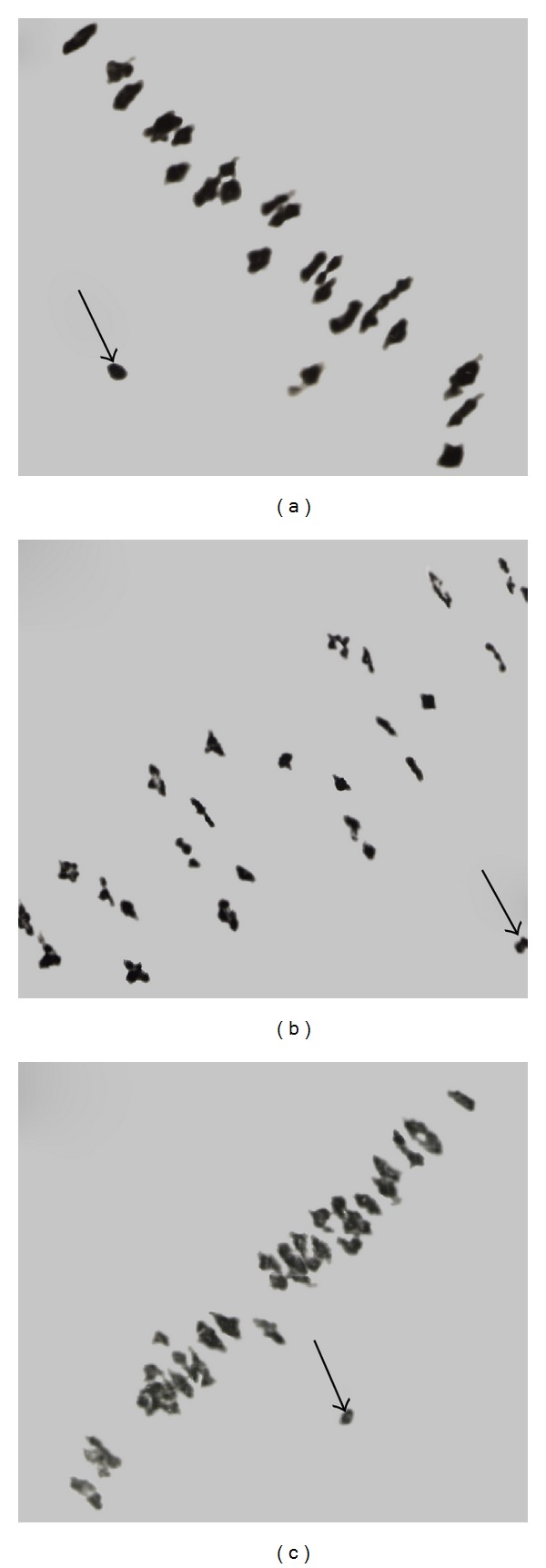
Meiotic configurations in primary monosomics in cotton *G. hirsutum*. Meiotic metaphase-I cells showing 25 bivalents and 1 univalent in (a) Mo71 (medium univalent size); (b) Mo16 (large univalent size); (c) Mo80 (small univalent size). The arrows point to the univalents. Note that the background of figures was cleaned using Adobe Photoshop CS 2 version 9.0.

Analyses of the sizes of monosomes revealed medium univalent size in 43 monosomics (Figure 2(a)), whereas there were 21 monosomics with large univalents (Figure 2(b)). The number of monosomics having small univalents was slightly higher (27); moreover, among these, 6 monosomics with very small univalents were detected (Figure 2(c)). Therefore, according to a preliminary assignment of monosomes on the basis of their sizes to the subgenomes, 21 large monosomes can be assigned to the A_t_-genome and 27 monosomes of small sizes to the D_t_-genome. Since it is known that only 3 chromosome pairs of *G. hirsutum* have long arms that are two or three times the length of the short arms [29], monosomes of medium sizes demand special analyses using translocations with subgenome assigned interchanges. It should be mentioned that studies of subgenome assignment of unidentified monosomes of medium sizes showed the A_t_-subgenome location [28] and significant deviation from the expected 1 : 1 ratio of the A_t_-subgenome monosome number to the D_t_-subgenome ones. Independent analyses of the same monosomic stocks revealed that A_t _- versus D_t_-genome monosome number ratio varied from 5 : 1 [27, 30] to 1.7 : 1 [28]. The latter ratio was much smaller than those previously reported. These findings also implied that preferential loss of the A_t_-genome chromosomes was caused by specific genetic regulation system of chromosome disjunction and was not due to size of monosomes [28]. In our experiments, we detected a nearly 2 : 1 ratio of the A_t_- to the D_t_-genome monosomes if we regard monosomes of medium sizes to be from A_t_-genome. Our ratio is not significantly different from the ratio given by Myles and Endrizzi [28]. This confirms a greater tolerance of *G. hirsutum* to loss of the large A_t_-genome chromosome than the small D_t_-genome chromosomes.

### 3.3. Analyses of Tetrads of Microspores and Pollen Fertility in Cotton Monosomics

Analysis of tetrads was carried out for 87 primary monosomics of our collection. Most of the monosomics (73 or 83.91%) had a higher meiotic index (more than 90%) than that of the control plants (95.11 ± 0.46%). This indicates regular univalent chromosome disjunction. Fourteen of the monosomics (16.09%) were characterized with lowering of meiotic index from 89.32% (Mo74) to 68.32% (Mo4). Moreover, 10 of the monosomics had a smaller reduction of meiotic index (to 80%) than did two others (Mo4 and Mo16). These monosomics were induced in M_1_ generation after pollen *γ*-irradiation in doses of 20 and 25 Gy, leading to strong meiotic index reduction (to 68.32 ± 1.10% and 76.07 ± 0.93%, resp.). We also observe an increase of percentage of tetrads with micronuclei (to 6.87 ± 0.60% and 21.56 ± 0.90%, resp.) in comparison with the control line (1.42 ± 0.25%; Table 3). Two other monosomics (Mo88 and Mo90), selected from M_3_ generation after irradiation of seeds by thermal neutrons and pollen with *γ*-rays, respectively, were characterized with different meiotic index in various buds. Variation limits were also observed for the number of tetrads with micronuclei (Table 3).

Meiotic index decrease in 6 monosomics (Mo16, Mo28, Mo52, Mo74, Mo88, and Mo90) could be explained with the presence of additional univalents at meiotic metaphase-I. In contrast, meiotic index decrease in 4 monosomics (Mo8, Mo21, Mo23, and Mo57) was connected with simultaneous translocation heterozygosity that led to chromosome disjunction disturbances and the production of tetrads with micronuclei. However, meiotic index decrease in 3 monosomics with the modal chromosome pairing (Mo4, Mo34, and Mo37) and increase of number of tetrads with micronuclei in Mo4 (to 6.87 ± 0.60%) directly demonstrated disturbances in monosome disjunction and imbalanced gamete formation. However, unlike cotton, in wheat monosomics, many micronuclei were observed in tetrads that suggested high univalent misdivision rate [31, 32].

Monosomic plants with a reduced meiotic index also differed from each other by pollen fertility. Complete pollen sterility was recorded in two monosomics (Mo57 and Mo74); however, partial female fertility provided seed set from outcrossing. Such strong variability of pollen fertility in the monosomics with reduced meiotic index was explained with both cytogenetic status influence and specific radiation action especially in 8 monosomics isolated from M_1_ generation after irradiation.

**Figure 3:**
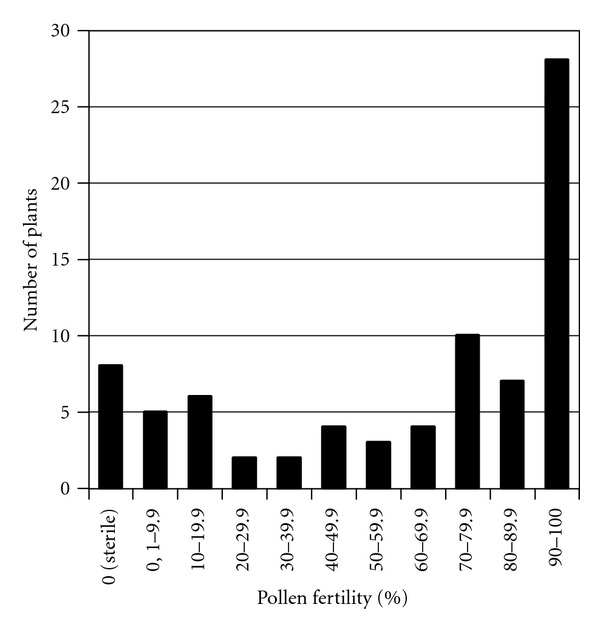
Percentage distribution of pollen fertility for 79 cotton monosomic plants. Note: the remaining 11 monosomics were not included in the histogram due to their varied pollen fertility level in different flowers.

Pollen fertility after acetocarmine staining was studied in 90 primary cotton monosomics isolated mainly from different types of irradiation. Although the acetocarmine-based pollen fertility considered relatively insensitive method, it is widely used (i.e., see [33]) for preliminary screening of pollen quality in plants. High pollen fertility was detected only in 28 plants with chromosome deficiencies that pointed out probable early haplo-deficient microspore abortion prior to mature pollen stage. Remaining monosomics were characterized with pollen fertility decrease. Thus, 17 monosomics had small reductions in pollen fertility (to 70%), 11—semisterile pollen (to 40%) and 15—strong pollen fertility reduction (to 5%). Pollen sterility was established in 6 monosomics (Mo5, Mo7, Mo10, Mo44, Mo45, and Mo47) derived from M_1_ generation after irradiation of pollen and in two monosomics (Mo57 and Mo74) isolated after thermal neutron seed irradiation (Figure 3). Monosomics Mo5 and Mo44 did not produce any seeds that suggest their complete sterility. In 11 monosomics, pollen fertility varied among different flowers on the same plant; moreover, the variation limits strongly differed. Reproduced monosomics from the 3 other families (Mo22, Mo39, and Mo46) also had pollen fertility variation in different flowers on the same plant from semisterile or low to reduced pollen fertility. 

Taken together, more than half of parental monosomics had partial or complete pollen sterility. After irradiation in M_1_ generation, pollen grain overabortion might be the result of radiation physiologic effect. However, transmission of the character to the next generations in daughter monosomics of higher generations (M_4_–M_6_) was obviously connected with gene(s) responsible for pollen development located in the monosome chromosomes (Mo22, Mo39, and Mo46). In due course, the abortive pollen detection in 6 monosomic lines suggested a polygenic control of pollen development in cotton *G. hirsutum* [34]. In addition, the high pollen fertility observed in some cotton plants with chromosome deficiencies can be due to certain tolerance to aneuploidy due to the polyploid origin of *G. hirsutum. *At the same time, failure to transmit the majority of cotton chromosome deficiencies via pollen [10] could be due to their negative effects on male gametophyte viability in general.

We used the individual seed weight method to facilitate monosome cytotype detection. According to Douglas' opinion [35], within a single boll, monosomic seeds are lighter than normal seeds. Douglas recommended use of this marker to increase the chance of revealing rarely transmitted monosomes. However, it was unknown whether the low seed weight was a universal marker of monosomy in cotton. To study the relationship of monosomic cytotypes and individual seed weight, we weighed each of the seeds in 32 monosomic lines. Our results indicate that light seed weight is not a universal marker of monosomy in cotton. In spite of the tendency to produce light seeds in 22 monosomics, some chromosome-deficient plants were transmitted from the seeds of medium weights (3 plants) and even heavy weights (3 plants). Similar reproduction features were also described for wheat monosomics of Diamant 2 variety [36].

### 3.4. Transmission Rates of Cotton Monosomics

The reproduction of the monosomics was studied in the selfed and outcrossed progenies under field and greenhouse conditions. Comparative analysis of the cotton monosomics reproduced in the field revealed stronger morphological differences in comparison with disomic sibs as well as monosomics reproduced in the greenhouse. As a result, monosomics were reproduced in 18 progenies under field conditions. However, because of limited vegetation period, we did not manage to analyze most of the progenies and determine exact transmission frequency in the field. Eleven of them were reproduced later under greenhouse condition whereas 7 monosomics (Mo22, Mo34, Mo36, Mo39, Mo45, Mo46, and Mo53) have not been reproduced at this time. Progenies of 77 different monosomics were studied in the greenhouse. Table 4 includes data on 45 monosomics, 19 of which were reproduced for the first time. Revised data are presented for 16 of 26 monosomics characterized in our previous study [14] to show the complete information on transmission rates of the monosomics from the Uzbek Cotton Cytogenetic Collection. All monosomic families strongly differed in number of plants studied and in only 14 families were all progenies cytologically examined for monosome transmission rate. This demonstrated a large variation in transmission rate from high (44.44% in Mo16 and Mo84) to very low (2.38% in Mo15). The highest transmission rate (from 30.43% in Mo72 to 44.44% in Mo16 and Mo84) was observed for 8 monosomics (Mo16, Mo31, Mo66, Mo71, Mo72, Mo77, Mo82, and Mo84). This suggested haplo-deficient gamete frequent transmission. On the other hand, 10 other monosomics (Mo3, Mo4, Mo9, Mo15, Mo35, Mo40, Mo56, Mo61, Mo67, and Mo85) had the lowest transmission rate (from 2.38% in Mo15 to 9.38% in Mo67) due to rare *n* − 1 gamete transmission that demanded a large population study for their recovery. The remaining 29 monosomics were transmitted with medium frequencies (from 14.29% in Mo10 and Mo74 to 29.17% in Mo62). Significant variability in transmission rates could be explained by differences in the viability of haplo-deficient gametes involving specific chromosomes. Theoretically, after selfing, monosomics must produce progenies with 2*n*, 2*n* − 1, and 2*n* − 2 chromosome number in the ratio 1 : 2 : 1, but, in fact, cotton nullisomic gametes with 2*n* − 2 are nonviable whereas *n* and *n* − 1 gametes form in unequal frequencies because of lower haplo-deficient gamete viability and their uncompetitiveness in comparison with normal gametes. Thus, all the differences in detected transmission rates involve deficiencies in various chromosomes of the cotton genome. Nevertheless, transmission rate similarities in some monosomes in our collection could indicate identities that should be explored.

Similarly, the rate of the transmission of the haplo-deficient gamete varied from low (4%) in a monosome 9 to high (49%) in a monosome 4 from the USA Cytogenetic Collection and 3 monosomics (for chromosomes 3, 9, and 16) usually transmitted with the lowest frequencies [10]. Male *n* − 1 gamete transmission was recorded only in two monosomes (Mono 4 and Mono 6) that often occurred as spontaneous deficiencies in natural populations. It should be mentioned that the low transmission rate of the monosome 9 could be partially explained by abnormal cytological behavior due to existence of a genetic factor that controls one or more metabolic processes specific to embryo sac mitoses, which ensures the normal disjunction of chromosomes [28].

### 3.5. Transmission of Chromosome Aberrations to the Monosomic Progeny

Deficiencies for one chromosome arm occurred in the progenies of seven monosomics. Thus, in three monosomic progenies (Mo17, Mo19, and Mo61) that differed with respect to monosome transmission rates (Table 4), monotelodisomics were produced due to univalent instability and resulted in misdivision. In the progenies of Mo21, Mo49, Mo54, and Mo68, daughter monosomics failed to produce, but monotelodisomics (from the progenies of Mo21, Mo49, and Mo68) and a monoisodisomic plant (from the progenies of Mo54) were detected. The results suggested an irregular univalent chromosome centromere misdivision in the parental monosomics that led to a single chromosome arm missing and formed either telocentric or isochromosome in the case of an arm doubling. Our results demonstrated the rather rare occurrence of telo- and isochromosomes in the monosomic progenies studied, which showed univalent misdivision to be rare. It should be mentioned that analysis of monosomic progeny from the USA Cytogenetic Collection revealed high misdivision rate in three monosomic plants—Mono12, Mono22, and Mono25 [9, 10]. However, according to Brown's opinion [37], univalents in cotton are seldom observed in meiosis. 

**Figure 4:**
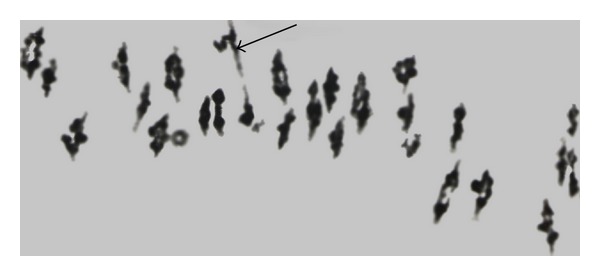
Meiotic metaphase-I in monotelodisomic plant from progeny Mo21, showing 25 normal bivalents and monotelodisomic bivalent (including one normal chromosome plus one telosome). The arrow indicates the monotelodisomic bivalent. Note that the background of figures was cleaned using Adobe Photoshop CS 2 version 9.0.

High heteromorphic bivalent frequency was observed in all of the six monotelodisomics (to 1.70 ± 0.11 in average per cell) suggesting telocentric chromosome pairing with the normal homologous chromosome (Figure 4). The various sizes of such bivalents preliminarily suggest that telosomes have different subgenome locations. Two telosomes involved long chromosome arms whereas the remaining involved short arm of cotton chromosomes. When telosome pairing with normal homologous chromosome is absent because of lower chiasmata frequency (especially in short-arm telocentrics), two univalents of different sizes form. Moreover, with telosomes involving short chromosome arms, more cells with univalents were observed (from 23.81% to 77.78% PMC) in comparison with the telosomes for long arms (from 12.60% to 14.29% PMC). In the monoisodisomic plant (from progeny Mo54), two unpaired chromosomes of different sizes (0.50 ± 0.15 in average per cell) were detected in 24% of PMCs. It was shown before that most of the pollen grains produced by PMCs with two univalents were deficient for chromosome 4 and nonfunctional [38]. Absence of frequent misdivision in the monosomics from Uzbek Cytogenetic Collection suggests monosome stability in the background of the line L-458 (genetic standard to development of monosomic lines in Uzbekistan). This monosome stability further is supported by the well-known phenomenon of coincidence of misdivision frequencies of centromers in monosomics with the frequency of iso-and telocentric chromosomes in their progenies. Moreover, this observation also implies the absence of the cotton monosomics for the chromosomes 12, 22, and 25. We cannot eliminate the influence of genetic background in this process. For example, in wheat genotype *(Triticum aestivum *L.*),* the role in univalent behavior was detected since the chromosome 5A misdivided more often in Chinese Spring background (39.7%) than in other varieties. The genotype of Chinese Spring variety proved to be favorable to high misdivision rate, and more than half of misdivided univalents produced isochromosomes [39].

Transmission in the progenies of 12 translocation heterozygous monosomics revealed daughter monosomic plants in only 6 progenies. Moreover, in two of them (Mo9 and Mo22), the daughter monosomics had no quadrivalents, and one progeny (Mo61) had heterozygous translocation, whereas two monosomics from the Mo63 and Mo54 progenies were translocation homozygotes. There were quadrivalent associations in F_1_BC_1_ hybrids from the crosses with standard line L-458. This suggests a positive role of the chromosome interchanges in these monosomics because of a selective advantage of the gametes containing single-chromosome deficiencies and the interchange between two other chromosomes in hetero- and homozygous conditions, respectively. 

According to a transmission study in 12 other monosomic families, disomic plants with desynaptic effect were detected due to desynapsis in parental plants and spontaneous desynaptic gene mutations in parental plants. Thus, analysis of monosomic progenies under field and greenhouse conditions provided daughter monosomic reproduction in 52 different families. However, the detection of other aberrations in 6 of them requires reanalysis of their progenies to isolate monosomics without any other karyotype disturbances.

The following selective behavior features were revealed in the cotton monosomics studied: karyotypic heterogeneity of progenies due to production of gametes with *n* and *n* − 1 chromosome set; various frequency of monosome transmission due to features of transmission of haplo-deficient gametes and their viability; low univalent misdivision frequency observed in a few products of monosome divisions; high stability of univalents in monosomics in the inbred line L-458 background; high reproduction rate of other types of mutations in progenies, originated in initial plants independently from monosomy.

## 4. Conclusions

We developed a new set of cotton monosomic stocks through radioactive irradiation of single genotype of L-458 cotton line. The results demonstrated detection of new unique desynaptic cotton plants in which progeny produced monosomics with high frequency. We observed the very rare occurrence of univalent misdivisions because of monosome stability in the unique genetic background. Our results demonstrate that light seed weight is not a universal marker for monosomy in cotton, and we detected possible univalent shifts in three monosomic progenies. Our observations with the development of reproductive organs of some monosomic plants suggested chromosome localization of genetic factors that control male gametophyte viability in the deficient chromosomes. Chromosomal identification of these new monosomic cotton stocks, using modern genetics methods (e.g., [11, 40–43]), is a high priority for future comparative studies of our collection with existing monosomic collections of cotton. For this effort, we are crossing our monosomic stocks with well-defined tester translocation line sets that were obtained from cytogenetic collection of the USA (kindly provided by Dr. D. M. Stelly and Dr. S. Saha through USDA-Uzbekistan cotton germplasm exchange program). Subsequent generation of monosomic-translocation hybrids will be used to locate monosomes for specific cotton chromosomes through cytogenetic analyses. Alternatively, the creation of chromosome substitution lines through crossing of each of the new monosomics with *G. barbadense* genotype (Pima 3-79) is in progress. This will serve as a foundation to apply molecular markers (e.g., SSRs) for the identification of our monosomics in hybrids with chromosome substitutions for a given monosome. At the same time, our monosomic cotton collection with initial cytogenetic characteristics, developed using single genome background, should be useful germplasm for cotton researchers to use as materials for future breeding, genetic, cytogenetic, and molecular-genetic investigations of the cotton genome.

## Figures and Tables

**Table 1:** The origin of the cotton primary monosomics from cytogenetic collection developed in Uzbekistan (radiation).

Dose of irradiation (Gy)	Number of primary monosomics*			Monosomic lines**
	M_1_	M_2_	M_3_	
Irradiation of seeds by thermal neutrons				

15	3	1	0	**—**
25	0	1	0	**—**
27	0	1	2	Mo1
35	1	2	0	Mo56, Mo62
Total	4	4	2	3

Irradiation of pollen by gamma rays				

10	5	4	2	Mo10, Mo39, Mo40, Mo50, Mo81, Mo82
15	4	9	1	Mo3, Mo31, Mo53
20	11	8	3	Mo4, Mo7, Mo11, Mo22, Mo27, Mo28, Mo34, Mo35, Mo36, Mo66, Mo75, Mo89
25	14	3	0	Mo9, Mo13, Mo15, Mo16, Mo17, Mo19, Mo38, Mo46, Mo48, Mo76, Mo77
Total	34	24	6	32

*A total number of primary monosomics in Table 1 is 74 that were developed using thermal neutron (10 monosomics) and gamma ray (64 monosomics) treatments. Remaining 18 primary monosomics were developed from desynaptic plants that were shown in Table 2. **From these 74 primary monosomics developed using irradiation, 34 monosomic lines were developed, and this number does not reflect the fertility or transmission ability of primary monosomics.

**Table 2:** Chromosome pairing at meiotic metaphase-I observed in PMCs and pollen fertility in the cotton desynaptic parental plants (DPPs) and their monosomics (Mo) progenies.

Material	Mo	Chromosome number	Chromosome associations				Pollen fertility	
			Total number of cells	Number of univalents	Frequency of chromosome associations (in average per cell)		Total number of pollen grains	Fertility, (%)
					univalents	bivalents		
**1609/6_6_-DPP**		**52**	**60**	**2–12**	**4.77 ± 0.48**	**23.62 ± 0.24**	**656**	**70.12 ± 1.79**
1609/6_6_-22	Mo55	51	22	1–3	1.45 ± 0.18	24.77 ± 0.09	466	93.99 ± 1.10
1609/6_6_-4	Mo69	51	33	1	1.00 ± 0.00	25.00 ± 0.00	499	98.27 ± 0.58
**1063/6_3_-13-DPP**		**52**	**42**	**2–28**	**14.33 ± 0.9**	**18.83 ± 0.45**	**405**	**65.68 ± 2.36**
1063/6_3_-13_3_	Mo70	51	25	1–3	1.08 ± 0.08	24.96 ± 0.04	639	95.15 ± 0.85
1063/6_3_-13_4_	Mo71	51	24	1	1.00 ± 0.00	25.00 ± 0.00	352	96.02 ± 1.04
1063/6_3_-13_5_	Mo72	51	21	1	1.00 ± 0.00	25.00 ± 0.00	632	96.87 ± 0.69
1063/6_3_-13_6_	Mo73	51	30	1–3	1.47 ± 0.15	24.77 ± 0.08	612	98.53 ± 0.49
**1570/14_9_-3-DPP**		**52**	**42**	**2–8**	**2.43 ± 0.31**	**24.79 ± 0.15**	**440**	**94.09 ± 1.12**
1570/14_9_-3_18_	Mo78	51	18	1	1.00 ± 0.00	25.00 ± 0.00	**—**	**—**
**1570/14_9_-13-DPP**		**52**	**15**	**2–6**	**2.67 ± 0.46**	**24.67 ± 0.23**	**467**	**95.29 ± 0.98**
1570/14_9_-13_7_	Mo85	51	16	1	1.00 ± 0.00	25.00 ± 0.00	984	97.97 ± 0.45
**179/2-DPP**		**52**	**32**	**2–8**	**2.50 ± 0.37**	**24.75 ± 0.19**	**157–537**	**2.61 ± 1.27–91.81 ± 1.18**
179/2_12_	Mo87	51	20	1	1.00 ± 0.00	25.00 ± 0.00	217–693	40.09 ± 3.33–92.93 ± 0.97
**356/8-DPP**		**52**	**18**	**2**	**1.67 ± 0.18**	**25.17 ± 0.09**	**401**	**61.35 ± 2.43**
356/8_5_	Mo58	51	33	1–3	1.12 ± 0.08	24.94 ± 0.04	685	81.02 ± 1.50
356/8_6_	Mo59	51	24	1	1.00 ± 0.00	25.00 ± 0.00	222–1292	2.25 ± 1.00–70.12 ± 1.27
356/8_7_	Mo60	51	22	1	1.00 ± 0.00	25.00 ± 0.00	307	38.44 ± 2.78
**356/8_8_-unknown caryotype**		**—**	**—**	**—**	**—**	**—**	**452**	**53.98 ± 2.34**
356/8_8_-14	Mo79	51	22	1	1.00 ± 0.00	25.00 ± 0.00	666	92.94 ± 0.99
356/8_8_-15	Mo80	51	20	1	1.00 ± 0.00	25.00 ± 0.00	447	99.78 ± 0.22
356/8_8_-5	Mo84	51	39	1–3	1.41 ± 0.13	24.79 ± 0.07	322	98.14 ± 0.75
356/8_8_-2	Mo91	51	19	1–3	1.11 ± 0.11	24.95 ± 0.05	65–726	53.85 ± 6.18–89.81 ± 1.12

DPP: desynaptic parental plants showed in bold-faced letters.

**Table 3:** Analyses of tetrads and pollen fertility in cotton primary monosomics (Mo) with reduced meiotic index.

Mo	Dose of irradiation, (Gy)	Microsporocytes			Pollen fertility	
		Total number of microsporocytes	Meiotic index*	Tetrads with micronuclei, %	Total number of pollen grains	Fertility, %
Mo4	20	1777	68.32 ± 1.10	6.87 ± 0.60	475	93.47 ± 1.13
Mo6	20	1654	87.85 ± 0.80	2.48 ± 0.38	207	74.40 ± 3.03
Mo8	15	695	80.29 ± 1.51	2.01 ± 0.53	593	61.72 ± 2.00
Mo16	25	2110	76.07 ± 0.93	21.56 ± 0.90	185	5.43 ± 1.67
Mo21	15	2129	89.24 ± 0.67	5.87 ± 0.51	387	18.58 ± 1.98
Mo23	20	765	88.76 ± 1.14	8.37 ± 1.00	132	81.82 ± 3.36
Mo28	20	858	87.18 ± 1.14	2.10 ± 0.49	450	70.67 ± 2.15
Mo34	20	1981	88.54 ± 0.72	2.68 ± 0.36	358	46.23 ± 2.64
Mo37	20	1317	81.85 ± 1.06	0.91 ± 0.26	185	82.16 ± 2.81
Mo52	15	1326	89.00 ± 0.86	2.56 ± 0.43	135	72.60 ± 3.84
Mo57	15	2684	87.15 ± 0.65	3.95 ± 0.38	325	0
Mo74	15	2295	89.32 ± 0.64	4.44 ± 0.43	340	0
Mo88	27	972–1785	47.94 ± 1.60/95.52 ± 0.49	0.62 ± 0.25/0.22 ± 0.11	983	91.96 ± 0.87
Mo90	20	203–2068	12.32 ± 2.31–98.69 ± 0.25	6.90 ± 1.78–0	2326	93.98 ± 0.49
L-458	0	2190	95.11 ± 0.46	1.42 ± 0.25	3200	96.44 ± 0.33

L-458 is original parental (control) genotype; meiotic index is a percentage of normal tetrads in all sporad.

**Table 4:** Transmission of the monosomes in the progenies of cotton monosomics (Mo) under greenhouse conditions.

Mo	Total no. of plants	No. of studied plants	Disomics (26II)	Monotelodisomics (25II+1t)	Monosomics (25II+1I)	Transmission (%)	No. of progenies
Transmission of monosomes studied in outcrossed progenies							

(Mo1)*	6	6	5	0	1	16.67	1
(Mo2)	6	6	5	0	1	16.67	1
Mo3	29	24	23	0	1	4.17	3
Mo4	45	29	27	0	2	6.9	2
(Mo10)	7	7	6	0	1	14.29	2
Mo11	52	34	24	0	10	29.41	4
Mo13	27	21	18	0	3	14.29	3
Mo15	44	42	41	0	1	2.38	5
(Mo28)	5	5	4	0	1	20.00	1
Mo56	26	26	24	0	2	7.69	4
Mo63	19	13	11	0	2	15.38	2
(Mo74)	12	7	6	0	1	14.29	1

Transmission of monosomes studied in selfed progenies							

Mo7	29	19	14	0	5	26.32	2
Mo9	48	34	32	0	2	5.88	3
Mo16	22	18	10	0	8	44.44	3
Mo17	33	31	24	1	6	19.35	9
Mo19	38	31	24	1	6	19.35	4
(Mo27)	9	9	7	0	2	22.22	2
Mo31	25	25	16	0	9	36.00	5
Mo35	24	23	21	0	2	8.70	3
Mo38	17	17	14	0	3	17.65	2
Mo40	33	33	32	0	1	3.03	2
Mo42	30	25	21	0	4	16.00	2
(Mo48)	11	11	9	0	2	18.19	3
Mo50	37	26	20	0	6	23.07	3
Mo60	16	14	10	0	4	28.57	3
Mo61	41	18	16	1	1	5.56	3
Mo62	61	24	17	0	7	29.17	4
Mo66	31	31	20	0	11	35.48	3
Mo67	40	35	32	0	3	9.38	4
Mo69	18	18	13	0	5	27.78	2
Mo70	18	18	15	0	3	16.67	3
Mo71	20	20	13	0	7	35.00	2
Mo72	23	23	16	0	7	30.43	2
Mo73	28	24	18	0	6	25.00	2
Mo75	35	15	11	0	4	26.67	3
Mo76	31	18	14	0	4	22.22	4
(Mo77)	22	10	6	0	4	40.00	1
Mo79	31	22	16	0	6	27.27	3
Mo80	48	20	17	0	3	15.00	3
Mo81	21	12	9	0	3	25.00	2
(Mo82)	17	11	7	0	4	36.36	2
Mo84	31	18	10	0	8	44.44	2
Mo85	48	26	25	0	1	3.85	2
Mo89	47	21	17	0	4	19.05	3

*Families shown in parenthesis are too small to provide a very informative assessment.
